# Endocannabinoid System and TRPV1 Receptors in the Dorsal Hippocampus of the Rats Modulate Anxiety-like Behaviors

**Published:** 2012

**Authors:** Elham Hakimizadeh, Shahrbanoo Oryan, Akbar Hajizadeh moghaddam, Ali Shamsizadeh, Ali Roohbakhsh

**Affiliations:** 1 *Physiology-Pharmacology Research Centre, Rafsanjan University of Medical Sciences, Rafsanjan, Iran *; 2 *Department of Biology, Science and Research Branch, Islamic Azad University, Tehran, Iran*; 3 *Department of Biology, Faculty of Basic Sciences, University of Mazandaran, Babolsar, Iran*

**Keywords:** Anandamide, Anxiety, CA1 Region, Fatty-acid amide hydrolase, Rats, TRPV Cation Channels

## Abstract

**Objective(s):**

Fatty acid is amide hydrolase which reduce endogenous anandamide. Transient receptor potential vanilloid-1 (TRPV1) channels have been reported to have a role in the modulation of anxiety-like behaviors in rodents. In the present study, the effects of either endocannabinoid system or TRPV1 channels and their possible interaction on anxiety-like behaviors of the rats were explored.

**Materials and Methods:**

Elevated plus-maze test of anxiety was used to induce anxiety. Capsaicin and AMG 9810 as TRPV1 agonist and antagonist respectively were injected into the dorsal hippocampus. URB 597 as selective FAAH inhibitor and AM 251 as CB1 receptor selective antagonist were also injected into the dorsal hippocampus. The effect of AMG 9810 on the response of URB 597 was also examined.

**Results:**

Intra-CA1 injection of URB 597 (0.001, 0.01 and 0.1 µg/rat) and AMG 9810 (0.003, 0.03 and 0.3 µg/rat) produced anxiolytic-like effects. Intra-CA1 infusion of capsaicin (0.003, 0.03 and 0.3 µg/rat) increased the anxiety-related behaviors and AM 251 (0.001, 0.01 and 0.1 µg/rat) did not significantly change the animals behavior. AMG 9810 at the dose of 0.003 µg/rat did not change the anxiolytic-like effect of URB 597.

**Conclusion:**

The results of the present study demonstrated that both endocannabinoid system and TRPV1 receptors may affect anxiety-like behaviors. In addition, it seems that TRPV1 receptors are not involved in the effects of anandamide on anxiety-related behaviors in the CA1 region.

## Introduction

The endocannabinoid system has a modulatory role in several physiological processes, particularly in the brain (1, 2). Studies suggest that by activating cannabinoid CB1 receptors, endocannabinoids play an important role in the control of anxiety (3). Anandamide is the best recognized of the endocannabinoids isolated so far. It is hydrolyzed within cells by fatty acid amide hydrolase (FAAH) (4). Therefore, selective inhibitors of FAAH enzyme such as URB 597 can magnify and prolong the duration of action of endogenously released anandamide. The anxiolytic-like effects of URB 597 in the isolation-induced ultrasonic vocalizations in rat pups and in adult rats tested in the elevated zero maze have been revealed (5). 

Transient receptor potential vanilloid-1 (TRPV1) channel is a receptor that is activated by capsaicin, a pungent component of hot chili peppers, as well as by low pH and noxious heat (6). These receptors are involved in pain perception, regulation of body temperature, bladder function, itching and pulmonary diseases [for review see Moran *et al* (7)]. Furthermore, TRPV1 receptors are expressed in brain regions associated with control of emotional and stress responses including the hippocampus, medial prefrontal cortex, locus coeruleus and dlPAG (dorsolateral periaqueductal grey) (8, 9). However, the role of TRPV1 receptors in the modulation of anxiety-like behaviors has not been explored well so far. In 2004, Kasckow *et al* (10) provided the first evidences on the anxiogenic-like effect of olvanil (TRPV1 receptor agonist) and the anxiolytic-like effect of capsazepine (selective TRPV1 receptor antagonist). These findings have also been repeated in some recent studies. 

Until now, the selective and exclusive endogenous ligand (s) for TRPV1 receptors has not been discovered. However, it is now apparent that anandamide is also an endogenous ligand for these receptors (11). Although, CB1 and TRPV1 receptors are expressed in several brain regions but they may have opposite roles in the regulation of neural activity (11). CB1 is a receptor which inhibits adenylate cyclase and reduces neurotransmitter release in the central nervous system (12) but TRPV1 receptor promotes depolarization of the neurons (13). Thus, anandamide may interact with CB1 and TRPV1 receptors to inhibit or promote anxiety-like behaviors, respectively. The functional consequences of the interaction between these receptors have rarely been explored (11). Hippocampus is a brain structure with an important role in the modulation of anxiety-related behaviors and a high density of CB1 and TRPV1 receptors and FAAH enzyme exist in this area (8, 14). 

On the basis of the above evidences, the present study was designed to investigate the effects of a FAAH inhibitor and selective TRPV1 agonist and antagonist on anxiety-like behaviors in the CA1 region of the hippocampus and to investigate a possible interaction between endocannabinoids and TRPV1 receptors.

## Materials and Methods

***Animals***

Male Wistar rats, weighing 200-250 g at the time of surgery, were used in the present study. Animals were housed 5 per cage, in a room with a 12:12 hr light/dark cycle (lights on 07:00 hr) and controlled temperature (23±2 ^o^C). Animals had access to food and water *ad libitum*. All rats were handled three days prior to the behavioral testing for 5 min. Each experimental group included seven animals. All experimental procedures were carried out according to the local Animal Ethics Committee protocols.

***Surgery***

Rats were anesthetized intraperitoneally with ketamine hydrochloride (75 mg/kg) and xylazine (4 mg/kg) and fixed in a stereotaxic frame. The stainless steel guide cannula (23-gauge) was implanted unilaterally in the right dorsal region of the hippocampus (CA1, coordinates: AP: -3.30 mm; L: 1.8 mm; D: -3 mm) according to Paxinos and Watson (15). It was then fixed to the skull with acrylic dental cement. 

***Drugs***

The drugs used in the present study consisted of AM 251 (selective CB1 receptor antagonist), capsaicin (TRPV1 receptor agonist), AMG 9810 (selective TRPV1 receptor antagonist) [Tocris, Bristol, UK] and URB597 (FAAH inhibitor) [Sigma Chemical, St Louis, MO, USA]. URB 597 and AM 251 were dissolved in DMSO and sterile 0.9% saline (up to 10% v/v) and five drops of Tween 80. AMG 9810 was dissolved in DMSO 100%. Capsaicin was dissolved in a mixture of Tween 80, ethanol and saline (1:1:8).

***Procedure***

Intra-CA1 injections were made using a microinfusion pump (KD Scientific, Holliston, MA, USA). Injector (30-gauge) was glued to polyethylene tubing filled with distilled water. The tubing was connected to a 10-μl microsyringe mounted on the microinfusion pump. Rats were hand-held as the experimenter inserted the injector. The injector projected 1 mm beyond the guide cannula and 0.5 µl solution was infused in the right CA1 region over a 60 sec period. The injector was left in place for an additional 60 sec to allow diffusion of the solution and to reduce the possibility of reflux. 

***Elevated plus-maze test of anxiety***

The method is mainly the same that was reported previously (16). The elevated plus-maze (EPM) comprised 2 open arms (50×10 cm) and 2 enclosed arms (50×10×40 cm) that extended from a common central platform (10×10 cm). An apparatus, constructed from wood, was elevated 50 cm above floor level. Testing was conducted in a quiet room between 9.00 a.m. and 13.00 p.m. Within 5 days following surgery, rats were brought into the behavioral testing room and left undisturbed for at least 1 hr prior to testing. The rats were individually placed in the center of the maze facing an open arm and allowed 5 min of free exploration. All sessions were videotaped. After each test, the floor was cleaned with distillated water. Animals were tested 5 min after microinjection of either AM 251, capsaicin or AMG 9810 and 30 min after microinjection of URB 597 (17). Thirty min is necessary to allow URB 597 to increase anandamide adequately in the CA1 region. Measurements were made from the frequencies of total open and closed arm entries (arm entry = all 4 paws into an arm) and the time spent in open, closed and central parts of the maze. The percentage of open arm entries (%OAE) and open arm time (%OAT) as the standard indices of anxiety-like behaviors were calculated. A significant decrease in the percentage of time in open arms and/or open arm entries is indicative of an increased level of anxiety. Total arm entries were measured as an index of locomotor activity (18).

***Experiments***

*Experiment 1*

**Figure 1:**
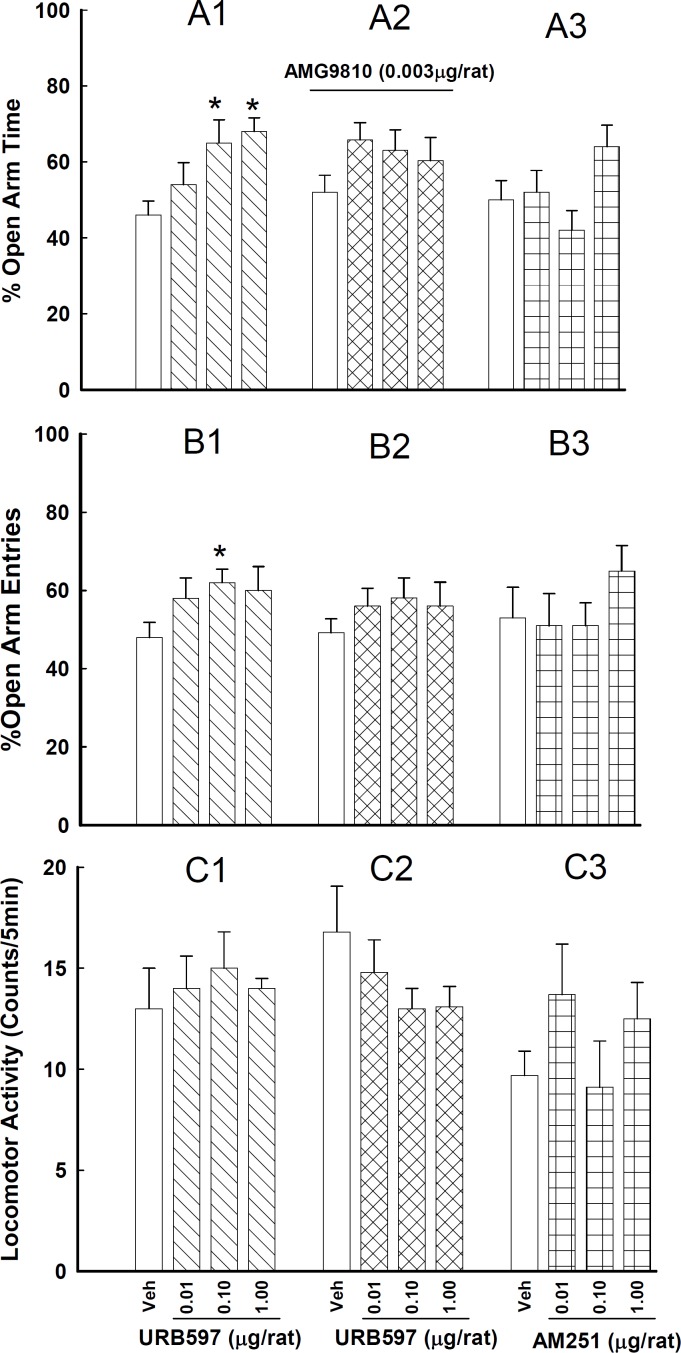
Effects of intra-CA1 injections of URB 597 alone (A1, B1, C1) or in the presence of AMG 9810 (A2, B2, C2) and the effects of AM 251 on anxiety-like behaviors (A3, B3, C3).

The animals received intra-CA1 injections of vehicle or one of the three doses of URB 597 [0.01, 0.1 and 1 µg/rat, doses based on Rubino *et al* (19)]. The test session was performed 30 min after intra-CA1 injections. %OAT, %OAE and locomotor activity were measured (Figure 1, A1, B1, C1).

*Experiment 2*

The animals received intra-CA1 injections of vehicle or one of the three doses of AM 251 [0.01, 0.1 and 1 µg/rat, doses based on Roohbakhsh *et al* (17)]. Five min after the injections, animals were submitted to the EPM test (Figure 1, A3, B3, C3).

*Experiment 3*

**Figure 2:**
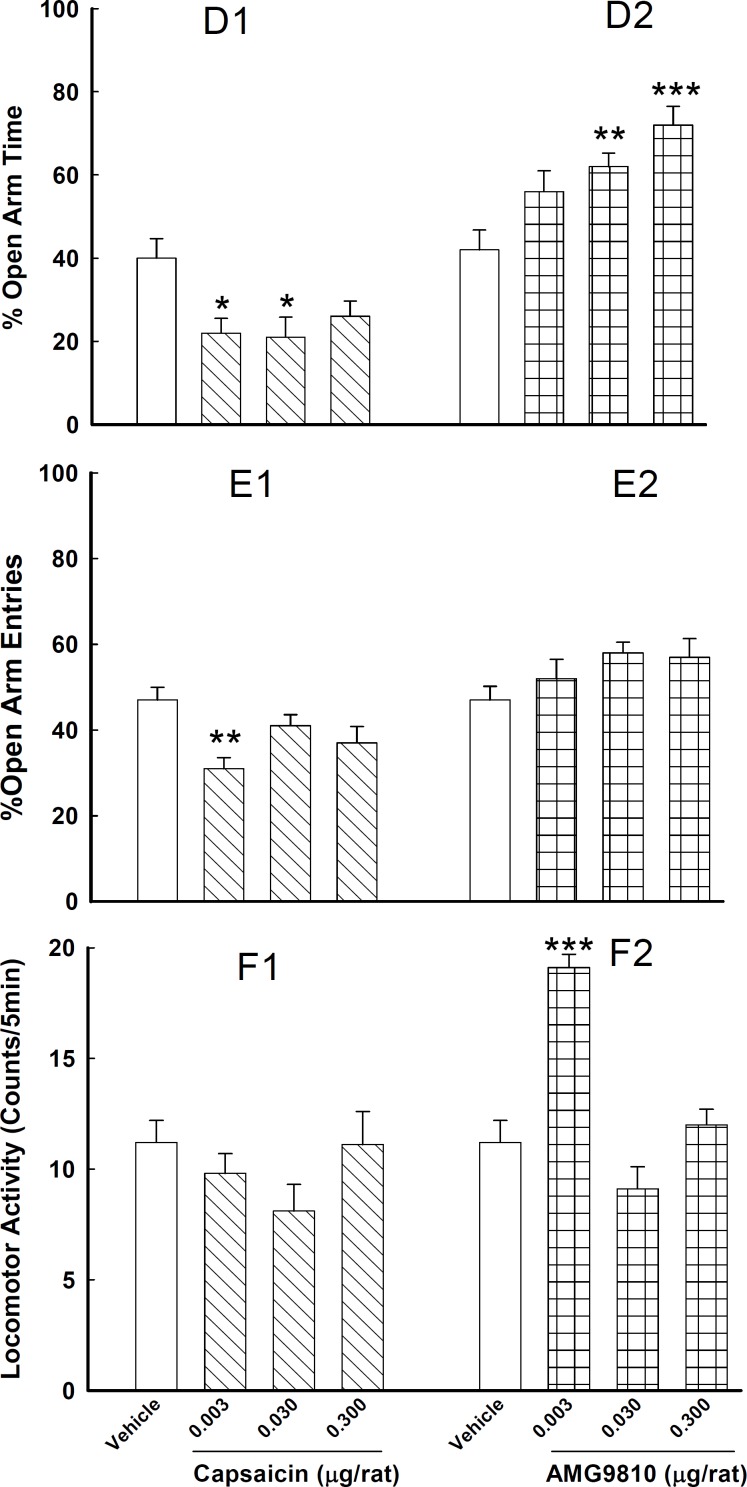
Effects of intra-CA1 injections of capsaicin (D1, E1, F1) or AMG 9810 (D2, E2 ,F2) on anxiety-like behaviors.

The animals received intra-CA1 injections of vehicle or one of the three doses of capsaicin [0.003, 0.03 and 0.3 µg/rat, doses based on Terzian *et al* (20)]. Five min after the injections, animals were submitted to the EPM test (Figure 2, D1, E1, F1).

*Experiment 4*

The animals received intra-CA1 injections of vehicle or one of the three doses of AMG 9810 [0.003, 0.03 and 0.3 µg/rat, doses based on Pitcher *et al* (21)]. 5 min after the injections, animals were submitted to the EPM test. (Figure 2, D2, E2, F2).

*Experiment 5*

The animals received intra-CA1 injections of vehicle or one of the three doses of URB 597 [0.01, 0.1 and 1 µg/rat]. After 25 min, the rats received an injection of AMG 9810 [0.003 µg/rat]. Five min after the second injections, the animals were exposed to the EPM test. (Figure 1, A2, B2, C2).

***Verification of cannula placements***

After completion of the experimental sessions, 0.5 µl of methylene blue was injected in the CA1 region of the rats. Ten min after the injections, the animals were decapitated and their brains were removed, blocked and cut coronally through cannula placements. Data from the rats with injection sites located outside the dorsal hippocampus region were not used in the analysis.

***Statistical analysis***

One-way ANOVA was used for the comparison between the effects of different doses of drugs with vehicles. Two-way ANOVA was used for evaluation of interactions between drugs. Following a significant F-value, *post-hoc* analysis (Tukey-test) was performed for assessing specific group comparisons. Differences with *P*< 0.05 between experimental groups at each point were considered statistically significant.

## Results

In the first experiment (Figure 1, A1, B1 ,C1), URB 597 at the doses of 0.1 and 1 µg/rat increased %OAT [*F*_(3,24)_ = 4.26, *P*< 0.05] and increased %OAE at the dose of 0.1 µg/rat [*F*_(3,24)_= 3.88, *P*< 0.05] without any significant change in the locomotor activity [*F*_(3,24)_= 0.22, *P*> 0.05]. This finding is suggesting an anxiolytic-like effect for URB 597.

Rats were injected either with vehicle (0.5 µl/rat) or URB 597 (0.01, 0.1 and 1 µg/rat) alone or AMG 9810 (0.003 µg/rat) 25 min after intra-CA1 injections of URB597. In another experiment, rats were injected with either vehicle (0.5 µl/rat) or AM 251 (0.001, 0.01 and 0.1 µg/rat). Each bar represents mean±SEM. n= 7. *: *P*< 0.05 when compared to the vehicle treated rats.

In the second experiment (Figure 1, A3, B3, C3), injection of AM 251 changed neither %OAT [*F*_(3,24)_= 2.69, *P*> 0.05] nor %OAE [*F*_(3,24)_= 0.88, *P*> 0.05] significantly. It means that AM 251 at these doses did not significantly affect animal behavior in the EPM. 

Rats were injected either vehicle (0.5 µl/rat) or capsaicin (0.003, 0.03 and 0.3 µg/rat) or AMG 9810 (0.003, 0.03 and 0.3 µg/rat). Each bar represents mean±SEM. n= 7. *: *P*< 0.05, **: *P*< 0.01 and ***:*P*< 0.001 when compared to the vehicle treated rats.

In the third experiment (Figure 2, E1, D1, F1), capsaicin decreased %OAT at the doses of 0.003 and 0.03 µg/rat [*F*_(3,24)_ = 4.20, *P*< 0.05] and decreased %OAE only at the dose of 0.003 µg/rat [*F*_(3,24)_= 5.70, *P*< 0.01]. Moreover, the changes in the locomotor activity was not significant [*F*_(3,24)_ = 1.44, *P*> 0.05]. The present finding suggests an anxiogenic-like effect for capsaicin in the EPM test of anxiety. 

In the forth experiment (Figure 2, E2, D2, F2), injection of AMG 9810 increased %OAT [*F*_(3,24)_ = 10.33, *P*< 0.001] at the doses of 0.03 and 0.3 µg/rat but not %OAE [*F*_(3,24)_= 1.91, *P*> 0.05]. An increase in the locomotion occurred at the dose of 0.003 µg/rat [*F*_(3,24)_= 25.28, *P*< 0.001]. These findings are suggesting an anxiolytic-like effect for AMG 9810 at the doses of 0.03 and 0.3 µg/rat.

In the fifth experiment (Figure 1, A2, B2, C2), the effects of co-administration of AMG 9810 (0.003 µg/rat) with URB597 on %OAT [*F*_ (3,48)_= 2.48, *P*> 0.05] and %OAE [*F*_(3,48)_ = 0.28, *P*> 0.05] was not significant when compared with the animals that received URB 597 alone. These finding is suggesting that TRPV1 receptors possibly are not involved in those effects of URB597 on anxiety-like indices in the CA1 region.

## Discussion

The main results of the present study demonstrated that both indirect activation of canabiniod receptors (by URB 597) and inhibition of TRPV1 receptors (by AMG 9810 ) in the CA1 region produced anxiolytic-like effects in the EPM while activation of TRPV1 receptors (by capsaicin) in this area induced anxiogenic-like effects. In addition, AMG 9810 did not change significantly the anxiolytic-like effects of URB597.

The current results showed that URB 597 produced anxiolytic-like effects in the CA1 region. In accordance with this finding, a number of previous studies have shown that inhibition of fatty acid amide hydrolase, the enzyme responsible for degradation of anandamide, induces anxiolytic-like effects in different animal models of anxiety. These findings have been reported following both peripheral (5, 22) and intra-prefrontal cortex (19) administration of URB 597. Kathuria *et al* (5), for the first time, showed that URB 597 had anxiolytic-like effects in mice without a typical spectrum of cannabinoid responses such as catalepsy, hypothermia or hyperphagia. Meanwhile, it has been reported that URB 597 has antidepressant-like effects (23) and despite common cannabinoids and anti-anxiety drugs increases wakefulness (24) and it does not have reward-related behaviors (25). Adding all these findings up, we may propose that this new class of drugs would be a good candidate for making a new generation of anti-anxiety drugs. However, injection of URB 597 in the ventral hippocampus has been documented with an anxiogenic-like response (17). This controversial effect has also been reported by Rubino *et al* (19) at a high dose of URB 597. These contradictory results may represent the importance of injection site and/or the doses of FAAH inhibitors that have been employed in different studies.

In the present study, injection of AM 251 into the CA1 did not produce any significant effect. In line with this finding, Moreira *et al* (26) found that intra-dorsal periaqueductal grey administration of AM 251 did not modify animal behaviors in the elevated plus-maze. The same results have also been reported following intra-ventral hippocampus and peripheral administration of AM 251 (17, 27). However, there are reports that have documented either an anxiogenic-like (28, 29) or an anxiolytic-like effect (30, 31) following administration of cannabinoid receptor antagonists. The above controversial results could be due to different methodological factors, sites of injections and different experimental conditions.

Our data showed that capsaicin, the active compound of hot chili, produced significant anxiogenic-like effects only at low and medium doses but not at the highest dose. A few numbers of the studies have evaluated the effect of TRPV1 agonists and antagonists on anxiety-like behaviors. Kasckow *et al* (10) showed that olvanil, a TRPV1 receptor agonist, induced anxiogenic-like effects in rats in the EPM test of anxiety. Conversely, capsazepine (a TRPV1 receptor antagonist) had anxiolytic-like effects in the same conditions. Rubino *et al* (19) also reported that infusion of capsaicin into the prefrontal cortex increased anxiety-like behaviors of the rats submitted to the EPM. In this study, capsaicin at the dose of 0.3 μg/rat (the highest dose) failed to show any significant change in animal behavior. Quick desensitization of TRPV1 receptors may be a possible explanation for this finding. Desensitization of TRPV1 receptors following administration of a high dose of capsaicin has been reported to be the reason for its analgesic effect after the initial hyperalgesia (32). This finding may suggest that TRPV1 receptors possibly have a modulatory role in the control of anxious states in the CNS. 

We also showed that injection of TRPV1 receptor antagonist (AMG 9810) induced anxiolytic-like responses. In agreement with the results of this study, the anxiolytic-like effect of capsazepine following intra-ventral hippocampus (33), intra-dlPAG (20) and intra-prefrontal cortex (19) of the rats has also been reported previously. Furthermore, TRPV1 knockout mice have shown reduced anxiety-like behavior and impaired fear conditioning in the light-dark and EPM tests of anxiety (34). It is noteworthy that the TRPV1 receptor antagonist that we used in this study has a better profile of receptor selectivity and greater antagonistic potency in comparison with capsazepine (35). This may give us a better deduction about the net effect of TRPV1 antagonists in the control of anxiety-like behaviors. The results also showed that AMG 9810 at the low dose of 0.003 μg/rat increased exploratory activity, as a possible confounding factor, of the rats. However, this increase did not happen at higher doses that decreased anxiety and the reason of this observation remains to be answered and we have no explanation for it.

It has been documented that anandamide is an endogenous ligand for both CB1 and TRPV1 receptors. It means that endocannabinoids by two different mechanisms may show their physiological roles in the body. This has been the topic of a number of studies. For example, the involvements of TRPV1 receptors in the effects of anandamide on blood pressure (36) and movement (37) have been reported. To test the hypothesis that TRPV1 receptors may be involved in those effects of anandamide in the modulation of anxiety-like behaviors in the CA1 region, we injected an ineffective dose of AMG 9810 on anxiety-like behaviors with three different doses of URB 597 as an indirect agonist of endocannabinoid system. Statistical analysis did not show any significant difference in animal behaviors in comparison with the rats that received URB 597 alone. In a recent study, Rubino *et al* (19) showed that pharmacological blockade of TRPV1 receptors by capsazepine reversed the anxiogenic effect of URB 597 in the prefrontal cortex of the rats. A possible explanation for this finding is that TRPV1 receptors are activated mainly at high concentrations of anandamide that induces anxiogenic responses. This hypothesis can be further supported by the results of Rubino* et al* (19) that showed the anxiolytic-like effect of methanandamide at low but not high doses is mediated by CB1 receptors. The bimodal action of anandamide on anxiety-like responses has been reported in a separate study (38). With this view, a new generation of anxiolytic drugs that have both FAAH inhibitory and TRPV1 antagonistic activity was developed recently (39). More experiments are necessary to elucidate the exact mechanisms underlying the effects of endocannabinoids in the modulation of fear or anxiety-like behaviors.

## Conclusion

FAAH enzyme inhibitors are a new class of drugs that may produce anxiolytic-like effects. These drugs increase wakefulness despite conventional anti-anxiety drugs and have a possible antidepressant effect that will increase their efficacy in clinical practice. Moreover, TRPV1 receptors may also have an important role in the regulation of anxiety-like behaviors and our knowledge about their interaction with endocannabinoids may help better production of more efficient anti-anxiety drugs.
